# Dimensional Accuracy After Precision Milling of Magnesium Alloys Using Coated and Uncoated Cutting Tools

**DOI:** 10.3390/ma17225578

**Published:** 2024-11-15

**Authors:** Jarosław Korpysa, Witold Habrat

**Affiliations:** 1 Department of Production Engineering, Mechanical Engineering Faculty, Lublin University of Technology, Nadbystrzycka 36, 20-618 Lublin, Poland; 2 Department of Manufacturing Techniques and Automation, Rzeszow University of Technology, Powstańców Warszawy 12, 35-959 Rzeszów, Poland; witekhab@prz.edu.pl

**Keywords:** precision milling, magnesium alloys, tool coating, dimensional accuracy

## Abstract

Magnesium alloys are an important group of materials that are used in many industries, primarily due to their low weight. Constantly increasing quality requirements make it necessary to improve the accuracy of manufactured products. In this study, the precision milling process for AZ91D and AZ31B magnesium alloys was investigated, and the results obtained with uncoated and TiB_2_-coated end mills were compared. The impact of variable cutting parameters was also investigated. Specifically, the study focused on the dimensional accuracy of the machined parts. The results showed that even though the dimensional accuracy obtained in milling both magnesium alloys was comparable, it was higher in the case of the AZ31B alloy by up to 22%. The study also demonstrated that the use of the TiB_2_ coating did not have the desired effect and that higher dimensional accuracy up to 27% was obtained with the uncoated tool.

## 1. Introduction

Precision machining is a key aspect of modern industrial manufacturing as it enables the production of high-quality products that meet the most stringent requirements. This is particularly important in the production of components that require strict compliance with design specifications [1]. In the aerospace and automotive industries, precision machining is used to manufacture precision mechanical parts, and in medicine to manufacture implants or surgical instruments. Although the problem of achieved accuracy is vital and constitutes the main goal of precision machining, it is unfortunately seldom analysed in scientific publications. This problem is predominantly considered in the context of the deformation of thin-walled parts [2,3,4,5] or elements with freeform surfaces such as blades [6,7], which are critical components primarily in the aerospace industry. Most studies usually investigate other indicators, such as surface roughness [8,9,10,11], cutting forces [12,13,14,15], and chips [16,17,18].

Manufacturing quality and accuracy have an impact on the mutual cooperation of machine components because, among other things, they ensure appropriate friction, which—in turn—affects the wear and service life of parts [19,20,21]. For this reason, it is vital to obtain surfaces with appropriate properties. The selection of a machining method depends on the product to be manufactured and the desired result. Milling is the most universal method that makes it possible to produce parts of any shape and complex geometry, especially nowadays with the increasing use of five-axis milling machines. However, studies [22,23] have shown that even higher accuracy could be achieved by wire-EDM machining. The application of this technique is unfortunately limited to machining parts of simple shapes. Nonetheless, better surface quality can be achieved through milling.

The quality of a finished product depends on many factors, such as the machine tool [24,25], workpiece fixture type [26,27,28], generated heat [29,30], deformations [31,32], machining time [33,34,35], among others. Unfortunately, these are mostly factors over which we have limited influence and are unable to change. However, there exist a few factors through which the machining process can be impacted in an effective way. These primarily include machining conditions, as any changes thereof can significantly affect the cutting process [36,37,38]. However, this requires prior research or relevant experience in this field. Admittedly, new and more advanced solutions are being developed to enable ongoing control and enhanced accuracy of manufactured parts, e.g., by changing the tool path based on continuous measurement of selected indicators. However, such systems are complex and require the use of special instruments and sensors for monitoring a wide array of factors in the machining environment [39,40,41,42]. This, in turn, is associated with high costs, which can pose a significant limitation on the application of these systems.

The cutting tool also plays an important role in the machining processes. Nowadays, cutting tools come in a variety of geometries and materials, which makes it possible to adapt them to a specific application. Protective coatings are also more and more often used to enhance the properties and lifetime of these tools [43]. This is because too rapid tool wear affects the accuracy of manufactured parts [44,45]. Coated tools are quite widely used for machining magnesium alloys. Marakini et al. [46] compared the milling process for the AZ91 magnesium alloy using tools with uncoated and TiN-coated inserts. Higher surface quality was obtained with the uncoated inserts, while the use of the coated inserts led to increased hardness. Changes in the machining parameters produced similar effects for both inserts. Surface roughness increased with increasing feed, while hardness decreased with decreasing cutting speed. The beneficial effects of using coated tools were also observed in the machining of the GW63K alloy [47]. The study was conducted using uncoated, TiAlN-coated, and DLC-coated end mills. Although the use of the TiAlN-coated tool slowed down tool wear, it also resulted in increased temperature and specific cutting energy. The TiAlN-coated tool produced the best results. Interestingly, the worst results were obtained with the DLC-coated end mill, which was due to adhesion. A comparison of different tool coats was also made in relation to the machining of Mg-SiC-B_4_C composites [48]. The use of a TiAlN-coated tool resulted in reduced tool wear and temperature rise while causing a considerable worsening of surface roughness. In contrast, a TiN-coated end mill produced the opposite effect. An end mill with TiCN-coated inserts was used to conduct tests on the AZ31 alloy [49]. The ANOVA results showed that reduced feed had the greatest effect on the Ra roughness parameter and led to a decrease in its value. Surface quality also improved when the cutting speed was increased and the depth of the cut was decreased. Similar relationships were also established in a study [50] that was conducted with the use of a Ti-NAMITE-coated end mill. The study also demonstrated that surface roughness improved with decreasing feed and increasing spindle speed. Changes in the machining parameters had the opposite effect on microhardness. In addition, the use of different cooling methods showed no clear changes. Research on the AZ31B alloy using coated inserts was also described in a study [51]. An increase in feed and cutting speed resulted in higher cutting force and temperature, as well as increased chip size.

The presented studies, however, focus on the conventional machining of magnesium alloys which differs significantly from precision machining. Given the fact that the number of studies and available publications on the precision machining of magnesium alloys is negligible, it is therefore necessary to broaden knowledge in this area and conduct new research in this field. The novelty of the current research is therefore that precision machining is performed on magnesium alloys. The analysis of dimensional accuracy is also an indicator quite rarely considered in scientific publications, even in relation to other light metal alloys. The aim of the study was to investigate the influence of cutting parameters and the application of a tool coating on the precision milling of selected magnesium alloys. The study focused on the analysis of the dimensional accuracy after machining. This allowed for the analysis of how the coating applied to the cutting tool and the adopted cutting parameters affect the technological aspect of machining accuracy. Since the precision machining process was carried out on a standard CNC machine, the obtained results have practical significance in relation to the finishing processes of magnesium alloys.

## 2. Materials and Methods

The study described in this paper focused on the implementation of a precision milling process for AZ91D and AZ31B magnesium alloys. These alloys were chosen due to their high industrial application. They belong to a group of materials with low density, high strength, and high vibration damping capability. They are widely used in the aerospace, automotive, and electronics industries due to their light weight and good mechanical properties. The mechanical properties and chemical compositions of both materials are given in Table 1 and Table 2.

**Figure 1:**
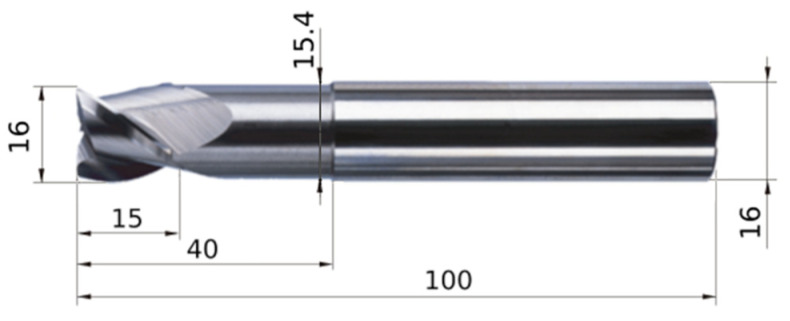
View of the cutting tool.

The milling process was conducted using 3-flute end mills with a diameter of 16 mm (see Figure 1). These were the AM3SSD1600A100 end mills from Mitsubishi (Tokyo, Japan). These tools are made of micro-grain WC-Co cemented carbide, whose particle size is less than 1 μm. This material structure is characterised by high hardness and transverse rupture strength. While both tools had the same geometry, one tool was uncoated and the other was TiB_2_-coated. In this way, it was possible to determine the effects of the application of tool coating. The coating was performed using the High-Power Impulse Magnetron Sputtering (HiPIMS) technology, which enables the application of coatings with a low thickness of only approx. 1–2 μm. In combination with the fine crystal structure of the material, it allows the sharp cutting edge to be maintained, which is so important in precision machining, while improving the tool’s properties. The coating reduces the tendency to build-up, reduces friction, and increases hardness by up to 5000 HV_0.05_. This coating also enables machining at temperatures of up to 1000 °C. Its main purpose is the machining of non-ferrous materials, and scientific papers confirm its beneficial properties and the advantages of its application [54,55]. The cutting edge radius *r_n_* of both tools was also determined prior to milling. It was 4.70 μm for the uncoated tool and 5.53 μm for the coated tool.

**Figure 2:**
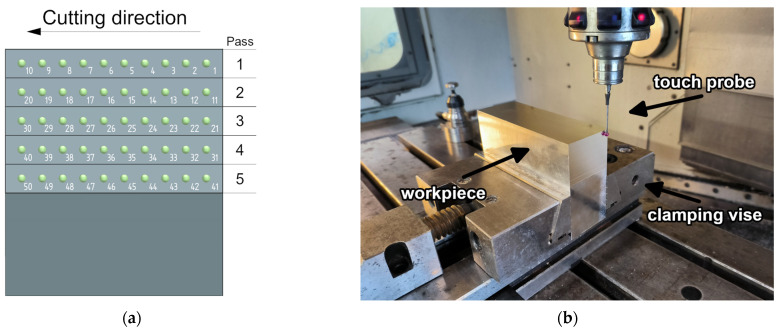
(**a**) Measurement scheme, (**b**) workspace view.

The study involved milling the side surfaces of the workpiece in the form of rectangular blocks with dimensions of 50 × 150 × 100 mm. The samples were clamped in a vice, allowing unrestricted surface machining. The milling operation was conducted in five tool passes with the axial depth of cut set to 7 mm. Measurements were taken at 10 points along each pass, according to the scheme shown in Figure 2. The measurements were performed immediately after the milling process using Heidenhain’s TS640 (Traunreut, Germany) touch probe that was mounted in the spindle of AVIA VMC 800HS (Warsaw, Poland). The obtained results were then used to determine the deviation from the assumed radial depth of cut and the range of these deviations. The deviation is defined as the difference between the theoretical value of the radial depth of cut and the value actually removed during machining.

Apart from tool coating, other variables investigated in this study included cutting parameters of the milling process. The cutting speed *v_c_* was varied in the range of 400–1200 m/min, the feed per tooth *f_z_* ranged from 1 to 9 μm/tooth, and the radial depth of cut *a_e_* from 60 to 100 μm. The effect of the variables on dimensional accuracy was also determined by ANOVA. The statistical analysis was performed using the Statistica 14 software.

## 3. Results

### 3.1. AZ91D Analysis

**Figure 3:**
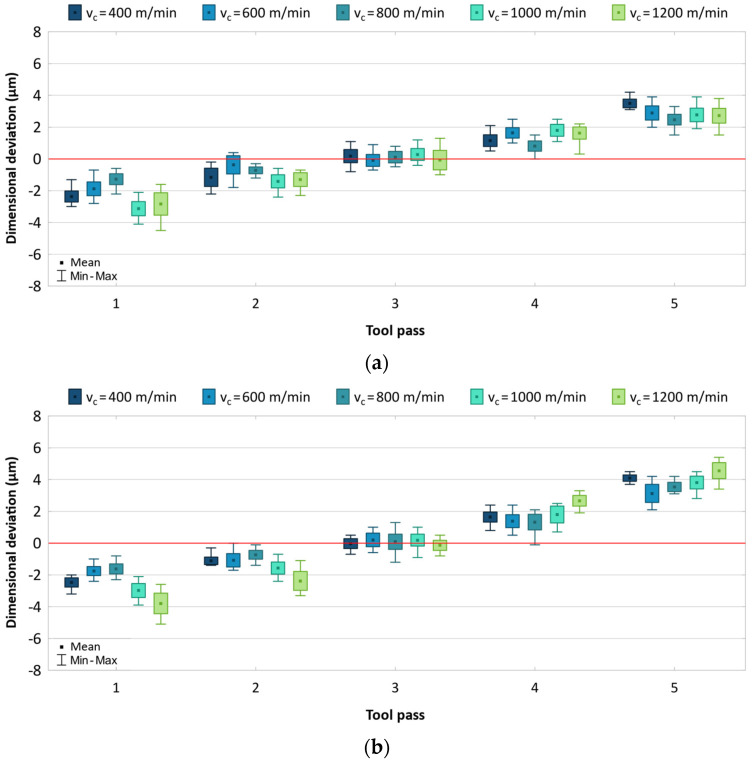
Results for different cutting speeds using (**a**) uncoated, (**b**) coated tool.

**Figure 4:**
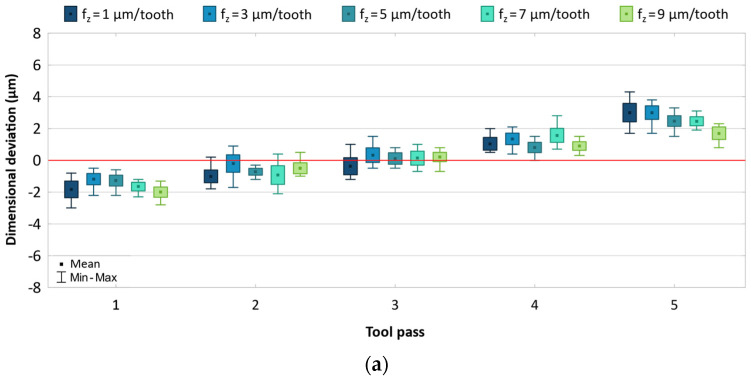

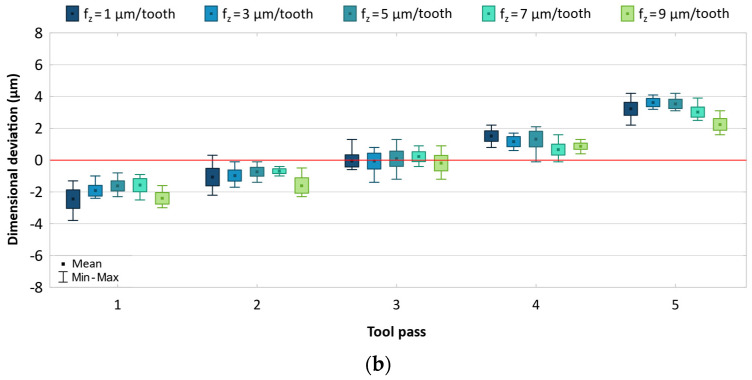
Results for different feed using (**a**) uncoated, (**b**) coated tool.

**Figure 5:**
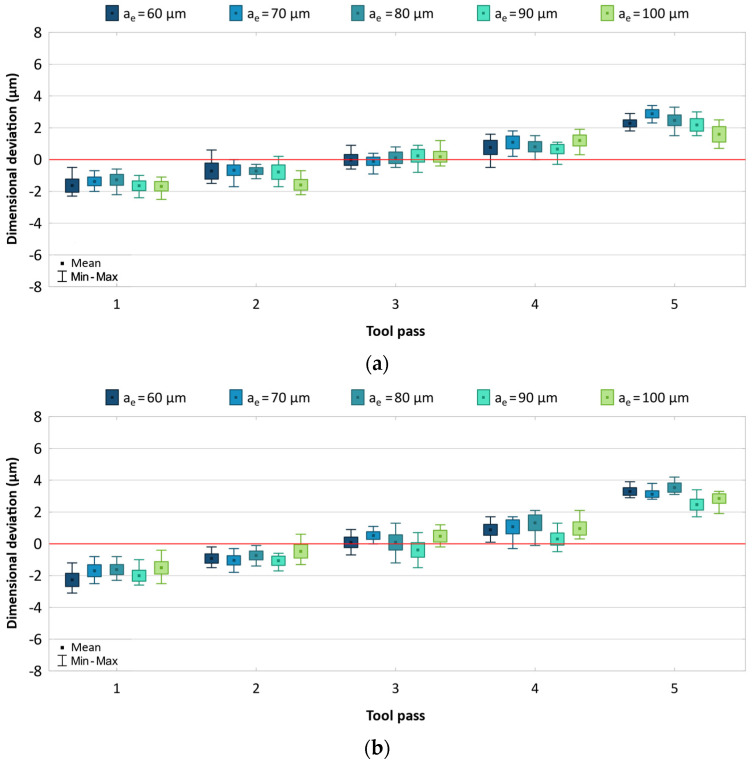
Results for different radial depths of cut using (**a**) uncoated, (**b**) coated tool.

The obtained results are presented as box-plots showing the real deviations of the surface dimensions from the assumed theoretical values. The results are analysed separately for each machining parameter and are given in Figure 3, Figure 4 and Figure 5.

During the machining of the side surfaces of the workpiece, the dimensional deviation gradually shifts with successive tool passes, with its value changing from negative to positive. This resembles the phenomenon of tool repulsion by the workpiece, which is rather unexpected given the relatively high rigidity of the cutting tools. One of the possible causes of this phenomenon is the potential occurrence of tool run-out, which results in the tool being pushed away from the machined surface. The results shown in Figure 3 confirm that the cutting speed change has a significant effect on the dimensional accuracy of produced parts. Regardless of the cutting tool used, the dimensional deviation range decreases until *v_c_* = 800 m/min, at which it reaches the minimum value and begins to increase again. For the machining process conducted with the uncoated tool, the dimensional deviation range changed from 5.5 to 8.3 μm, while for the process conducted with the TiB_2_-coated tool, it changed from 6.5 to 10.5 μm. The results obtained for both tools are also characterized by a similar scatter of values for individual machining passes.

A decrease in the feed per tooth leads to reduced dimensional accuracy of the parts (Figure 4). The dimensional deviation range for the milling process conducted with the uncoated and coated tools is 5.1–7.3 μm and 6.1–8.0 μm, respectively. As the feed is decreased, the scatter of values increases for individual tool passes. A longer machining time promotes the generation of higher temperatures in the cutting zone, which consequently leads to greater differences between the dimensions of produced parts. Reduced accuracy can also be associated with ploughing of the workpiece material. Nevertheless, the results show a fairly symmetrical distribution with respect to the zero line.

A change in the radial depth of cut has no significant effect on the dimensional accuracy of AZ91D magnesium alloy parts, but the effect of the cutting tool on the dimensional accuracy of AZ91D parts is considerable (Figure 5). A smaller dimensional deviation range of 5.0–5.5 μm is obtained when the machining process is conducted with the use of the uncoated tool. For the machining process conducted using the coated tool, the dimensional deviation range is 5.8–7.0 μm. Regardless of the tool used, a similar scatter of values is obtained for individual tool passes. For the machining process conducted with a variable radial depth of cut, one can also observe a uniform distribution of dimensional deviation with respect to the zero line.

**Figure 6:**
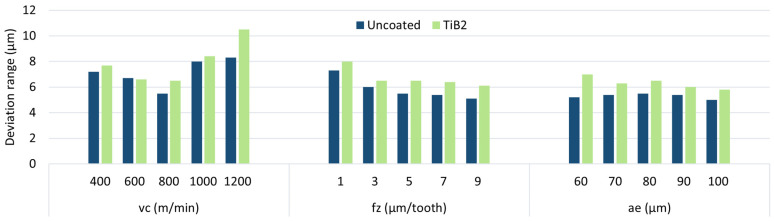
Deviation range for AZ91D magnesium alloy.

To better illustrate the effect of the machining parameters on the dimensional deviation range, the results are shown in a cumulative plot—Figure 6. It can be observed that the trend of changes is similar for both cutting tools, yet lower values are obtained with the uncoated tool. Nevertheless, the differences are relatively small. The greatest reduction in accuracy is observed for the machining process conducted with the highest cutting speed and the lowest feed. In contrast, no clear changes are observed when the radial depth of the cut is changed.

### 3.2. AZ31B Analysis

**Figure 7:**
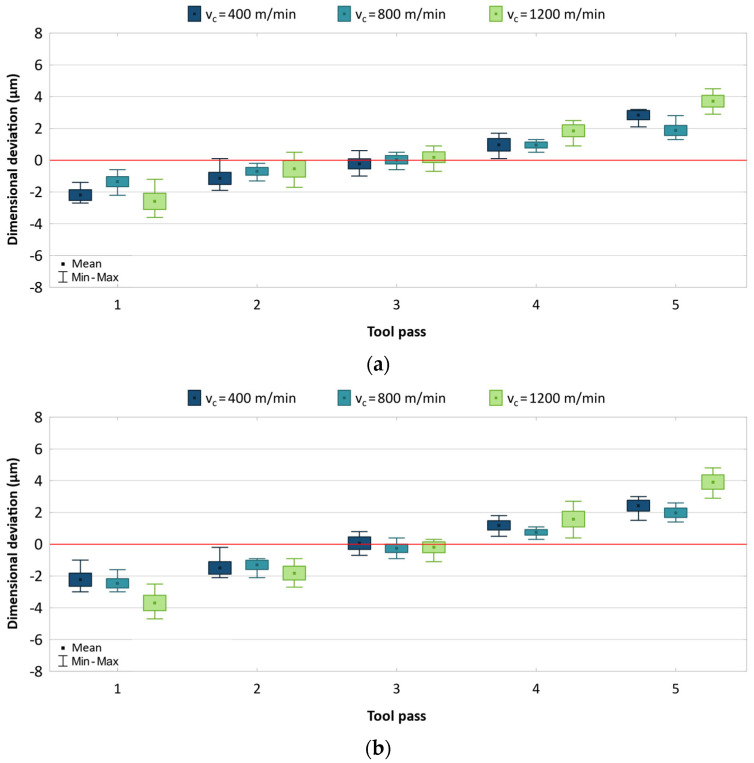
Results for different cutting speeds using (**a**) uncoated, (**b**) coated tool.

**Figure 8:**
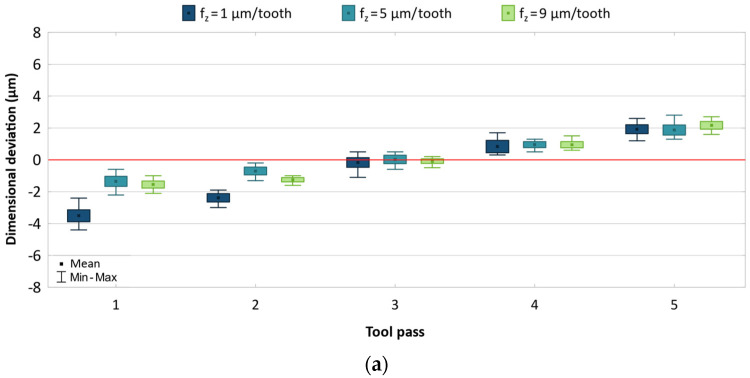

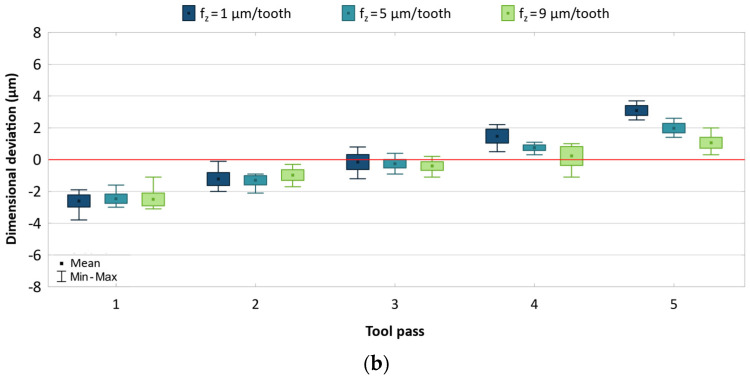
Results for different feed using (**a**) uncoated, (**b**) coated tool.

**Figure 9:**
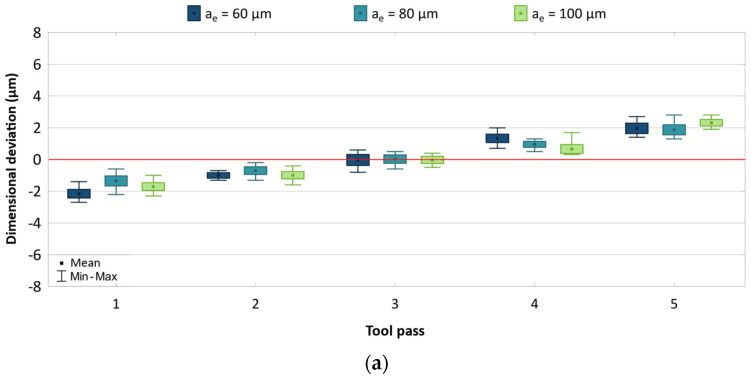

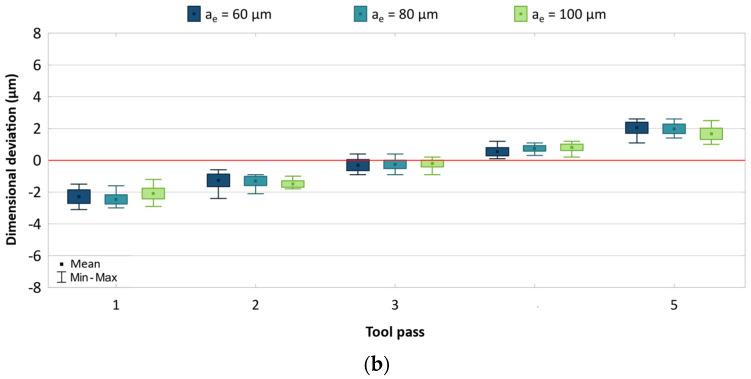
Results for different radial depths of cut using (**a**) uncoated, (**b**) coated tool.

A similar analysis was performed for the AZ31B magnesium alloy. The results of individual machining parameters are shown in Figure 7, Figure 8 and Figure 9.

When varying the cutting speed (Figure 7), a similar relationship can be observed as in the machining of AZ91D, which consists of increased accuracy at *v_c_* = 800 m/min. However, the dimensional deviation range obtained for the AZ31B alloy is smaller, especially at the lowest cutting speed, and is from 5.0 to 8.1 μm for the uncoated end mill and from 5.6 to 9.5 μm for the coated end mill. For both tools, there is a similar scatter of values at individual tool passes, and the results are distributed fairly symmetrically with respect to the zero line.

Figure 8 shows the effect of feed on dimensional accuracy. Like in the case of the AZ91D alloy, the dimensional deviation range decreases with increasing the feed, but the changes occur in a narrower range of 4.8–7.0 μm for the uncoated tool and 5.1–7.5 μm for the TiB_2_-coated tool. The reduced accuracy observed for the milling process conducted with low feeds can result from a more intense temperature increase in the cutting zone, as well as from the ploughing of the material. For most cases, a similar scatter of values is obtained for individual tool passes. Regardless of the cutting tool and feed change, the dimensional deviations are distributed approximately uniformly with respect to the zero line.

As for the milling process conducted with a variable radial depth of cut, no clear changes in dimensional accuracy can be observed (Figure 9). The type of end mill has no significant effect on the obtained results either. The dimensional deviation ranges are similar for both tools and amount to 5.0–5.4 μm and 5.4–5.7 μm for the uncoated carbide end mill and the TiB_2_-coated end mill, respectively. The scatter of values observed for individual tool passes is also on a comparable level, and the results are invariably characterized by a uniform distribution.

**Figure 10:**
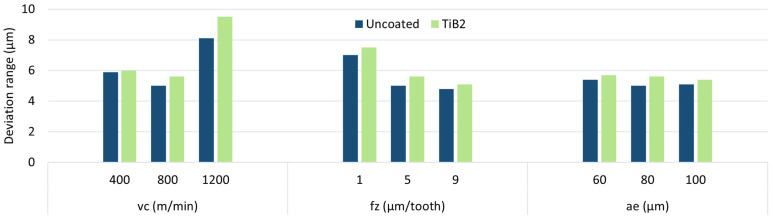
Deviation range for AZ31B magnesium alloy.

Figure 10 shows the dimensional deviation range in the milling process of magnesium alloy AZ31B parts. It can be seen that the changes made in the machining parameters had a similar effect to that observed for the AZ91D alloy. Again, the cutting speed and feed had the greatest impact on dimensional errors. The use of the uncoated end mill was again found to be more beneficial.

The milling of the side surfaces of parts showed several similarities between the tested materials. For both magnesium alloys, similar trends in dimensional accuracy changes due to changes in the technological parameters were observed, with the dimensional deviation ranges for individual tool passes being similar regardless of the tool type. However, a smaller scatter of values as well as a lower deviation range were obtained for most cases of AZ31B alloy machining, despite the higher ductility of this material. The use of the uncoated tool resulted in higher dimensional accuracy of AZ91D and AZ31B alloy parts, regardless of the changes in machining parameters.

### 3.3. ANOVA

The results obtained from the study were also subjected to a two-way ANOVA test (see Table 3, Table 4 and Table 5). The statistical analysis was carried out separately for AZ91D and AZ31B alloys due to different numbers of variance levels for the analysed machining parameters. The analysis was performed for a significance level of 0.05.

The ANOVA results showed that the changes in the machining parameters as well as the application of the tool coating had no significant effect on the mean value of the dimensional deviation (*p* > 0.05). This is related to the fact that despite their different values, the deviations are distributed approximately symmetrically, taking both positive and negative values. The mean value of the deviations obtained for all cases is therefore similar and equal to about zero. Therefore, ANOVA has not revealed a significant effect of the change in factors. The analysis showed that the only statistically significant interaction between the presence of a protective coating and feed change occurred in AZ31B magnesium alloy machining.

To determine the significance of the effect of machining parameters and coatings on dimensional deviations, a Levene test was performed (see Table 6). This test is used to examine the homogeneity of variances. It will therefore prove better for the obtained results, as deviation ranges are more important in this case than mean values. The test confirmed that changing the cutting speed and feed had a significant effect on the variances of the groups (*p* < 0.05), while the radial depth of cut was insignificant.

**Figure 11:**
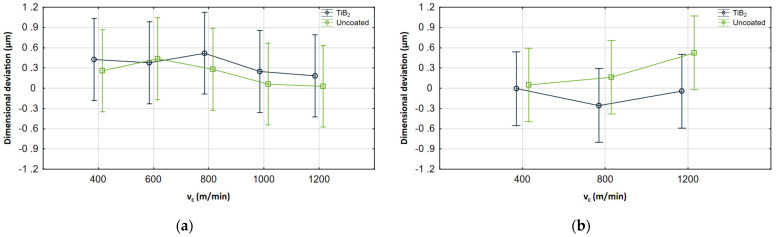
Interaction plots for *v_c_* (**a**) AZ91D, (**b**) AZ31B.

**Figure 12:**
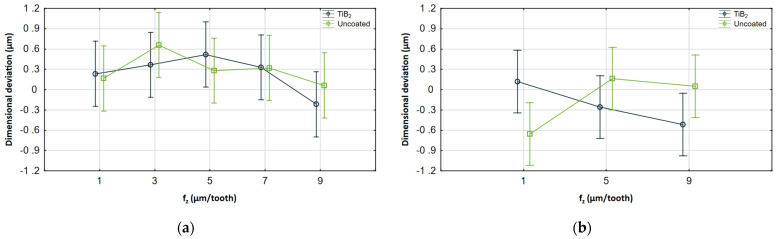
Interaction plots for *f_z_* (**a**) AZ91D, (**b**) AZ31B.

**Figure 13:**
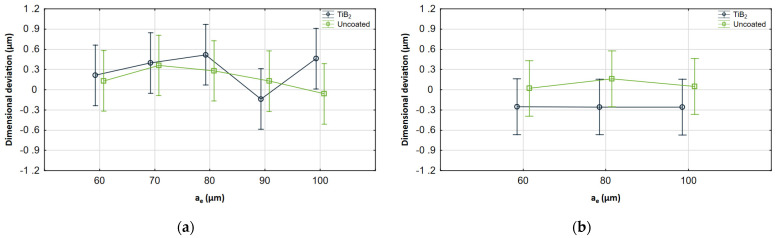
Interaction plots for *a_e_* (**a**) AZ91D, (**b**) AZ31B.

Interaction plots illustrating the ANOVA results are also presented in Figure 11, Figure 12 and Figure 13. For each cutting parameter, the cutting tool with and without coating was compared.

It can be seen that the results obtained for both tools are within similar ranges. Despite some differences resulting from changes in the machining parameters, the whiskers overlap in most of the common range, so the analysis showed no statistical significance. However, it is important to note that the analysis is based on the mean value and standard deviation, which reduces the impact of maximum deviations. Figure 12 also shows the change based on which the analysis revealed a significant interaction between coatings and feed. The results obtained for the uncoated tool in the milling process conducted with a feed of 1 μm/tooth differ significantly from the other results, which shows their significance. For other cases, the observed changes are considerably smaller.

## 4. Discussion

This study has demonstrated that changes in the machining parameters can have a significant effect on the dimensional accuracy of produced parts and that the effect of their change is similar for AZ91D and AZ31B alike. It has also been proven that the use of the TiB_2_-coated tool does not bring the expected improvement in the results and its application even negatively affected the achieved accuracy. An important observation from the study is that the dimensions change with successive passes of the cutting tool. This phenomenon is probably due to the heat generated in the cutting zone. The removal of an increasingly greater layer of the material with each tool pass indicates the heating up of both the tool and the workpiece material. Nevertheless, this does not explain the dimension changes observed for successive tool passes that would suggest that the end mill is pushed away from the workpiece, which leads to tool deflection. This is a rather unexpected phenomenon in view of the relatively high rigidity of the tools resulting from their small overhang-to-diameter ratio and low cutting forces acting on the tool. This type of phenomenon tends to occur when using cutting tools with low stiffness and accompanies the machining of thin-walled elements.

The results obtained are difficult to compare with other publications because, as mentioned in the Introduction Section, precision machining of magnesium alloys is a very new topic and only a few publications on the subject are available to date. Furthermore, none of these publications were concerned with accuracy measurements. It is generally a relatively rarely analysed indicator, also with respect to precision milling or micro-milling of other light metal alloys.

In the near future, research will be conducted on the effect of cutting zone temperature as it can cause the thermal expansion of workpieces and tools and thus lead to reduced manufacturing accuracy. If future studies indicate a significant impact of cutting temperature, then research into the application of cooling fluids will be reasonable. It also seems necessary to conduct research on cutting tool rigidity in order to verify whether this factor has any impact on resulting manufacturing inaccuracies.

## 5. Conclusions

The results of this study lead to the following conclusions:

- The milling process conducted on both tested magnesium alloys showed a gradual shift of dimensional deviations with successive tool passes, which may be due to the pushing away of the cutting tool from the previously machined surface of the workpiece.
- The dimensional accuracy of produced parts primarily depended on the applied cutting speed and feed per tooth, and in most cases, the changes in these parameters had similar effects, regardless of the magnesium alloy or tool coating. The highest dimensional accuracy was obtained for parts machined with the medium cutting speed and the highest feed. In contrast, the effect of the radial depth of cut was insignificant, which allows machining efficiency to be increased without worsening the accuracy of the components. This is particularly important when machining is performed with such low cutting parameters.
- It was observed that an increase in cutting speed to 1200 m/min caused a significant increase in deviation ranges, up to 52% for the AZ91D alloy and 61% for the AZ31B alloy. This may result from the increased effect of tool imbalance at higher spindle speeds or the increased temperature in the cutting zone resulting from high spindle speed. In the case of feed rate, increased deviation ranges up to 43–46% were observed for a feed of 1 μm/tooth, which may be the result of machining close to the minimum undeformed chip thickness. This is associated with a high probability of ploughing phenomenon, which deteriorates the condition of the machined surfaces. The dimensional changes may also be related to the thermal expansion of the materials since the very low feed rates mean the tool moves very slowly, generating heat almost in one area.
- Considering both magnesium alloys, better results were obtained when the milling process was conducted using the uncoated tool. The differences were relatively small (from 2 to 27%) but the coated end mill showed a tendency to larger deviation ranges. This may be related to an increase in the value of cutting edge radius and a change in the tribological conditions. In this case, the tested TiB_2_ coating did not show a beneficial effect on the machinability of both magnesium alloys.
- The AZ31B magnesium alloy parts exhibited higher dimensional accuracy. The deviation ranges obtained for this material were up to 22% lower compared to machining the AZ91D alloy with the same cutting parameters.
- Statistically, the cutting tool and technological parameters did not have a significant effect on the mean values of dimensional deviations, which resulted from obtaining mean values close to zero. However, in most cases, they had a significant effect on the variances. They therefore seem to be a more suitable indicator for this particular study.

Based on the obtained results, it is concluded that the improvement of dimensional accuracy is possible by changing the machining conditions. For this purpose, a medium cutting speed and high feed are strongly recommended, as this provides a lower dimensional deviation range. It is also advisable to increase the cutting width to improve machining efficiency. The use of an uncoated end mill is more advantageous due to better machining results and lower costs. The above suggestions concern the AZ91D and AZ31B magnesium alloys.

## Figures and Tables

**Table 1:** Standard mechanical properties of magnesium alloys [52,53].

Alloy	Hardness (HB)	Tensile Strength (MPa)	Yield Strength (MPa)	Elastic Modulus (GPa)	Elongation (%)
AZ91D	63	230	150	45	3
AZ31B	49	260	170	45	15

**Table 2:** Standard chemical composition of magnesium alloys [52,53].

Alloy	Al	Zn	Mn	Fe	Ni	Si	Others	Mg
AZ91D	8.3–9.7	0.35–1	0.15–0.50	0.005	0.002	0.1	0.02	Bal.
AZ31B	2.5–3.5	0.6–1.4	0.2	0.005	0.005	0.1	0.3	Bal.

**Table 3:** ANOVA results for *v_c_*.

Factor	AZ91D					AZ31B				
	SS	Df	MS	F	*p*	SS	df	MS	F	*p*
Coating	2.33	1	2.33	0.487	0.486	9.05	1	9.05	2.360	0.126
*v_c_*	8.00	4	2.00	0.419	0.795	4.49	2	2.24	0.585	0.558
Interaction	1.34	4	0.34	0.070	0.991	3.46	2	1.73	0.452	0.637
Error	2340.12	490	4.78			1127.27	294	3.83		
Total	2351.79	499				1144.27	299			

Sum-of-squares (SS), Degrees of freedom (Df), Mean squares (MS), F ratio (F), and significance (*p*).

**Table 4:** ANOVA results for *f_z_*.

Factor	AZ91D					AZ31B				
	SS	df	MS	F	*p*	SS	df	MS	F	*p*
Coating	0.33	1	0.33	0.110	0.741	0.35	1	0.35	0.128	0.720
*f_z_*	20.42	4	5.10	1.708	0.147	2.83	2	1.42	0.514	0.598
Interaction	5.32	4	1.33	0.445	0.776	27.02	2	13.51	4.908	0.008
Error	1464.34	490	2.99			809.30	294	2.75		
Total	1490.41	499				839.50	299			

**Table 5:** ANOVA results for *a_e_*.

Factor	AZ91D					AZ31B				
	SS	df	MS	F	*p*	SS	df	MS	F	*p*
Coating	1.89	1	1.88	0.725	0.395	8.30	1	8.30	3.777	0.053
*a_e_*	11.07	4	2.77	1.064	0.374	0.27	2	0.14	0.062	0.940
Interaction	8.34	4	2.08	0.801	0.525	0.29	2	0.14	0.066	0.936
Error	1274.57	490	2.60			646.01	294	2.20		
Total	1295.87	499				654.87	299			

**Table 6:** Levene’s test results.

Factor	AZ91D		AZ31B	
	F	*p*	F	*p*
*v_c_*	7.370	0.000	8.193	0.000
*f_z_*	2.798	0.003	9.228	0.000
*a_e_*	1.595	0.114	1.323	0.254

## Data Availability

The raw/processed data required to reproduce these findings cannot be shared at this time as the data also form part of an ongoing study.
